# Intra-Domain Cysteines (IDC), a New Strategy for the Development of Original Antibody Fragment–Drug Conjugates (FDCs)

**DOI:** 10.3390/pharmaceutics14081524

**Published:** 2022-07-22

**Authors:** Louis Jolivet, Imène Ait Mohamed Amar, Catherine Horiot, Fanny Boursin, Cyril Colas, Stéphanie Letast, Caroline Denevault-Sabourin, Emilie Allard-Vannier, Nicolas Joubert, Nicolas Aubrey

**Affiliations:** 1 ISP UMR 1282, INRA, Team BioMAP, Université de Tours, 31 Avenue Monge, 37200 Tours, France; louis.jolivet@etu.univ-tours.fr (L.J.); horiot.catherine@gmail.com (C.H.); fanny.boursin@univ-tours.fr (F.B.); nicolas.aubrey@univ-tours.fr (N.A.); 2 GICC EA7501, Team IMT, Université de Tours, UFR de Médecine, Bâtiment Vialle, 10 Boulevard Tonnelé, BP 3223, CEDEX 01, 37032 Tours, France; imene.aitma@gmail.com (I.A.M.A.); stephanie.letast@univ-tours.fr (S.L.); caroline.denevault@univ-tours.fr (C.D.-S.); 3 ICOA UMR 7311, Université d’Orléans, CNRS, Rue de Chartres, 45067 Orléans, France; cyril.colas@univ-orleans.fr; 4 CBM UPR 4301, CNRS, Université d’Orléans, Rue Charles Sadron, 45071 Orléans, France; 5 NMNS EA 6295, Université de Tours, 31 Avenue Monge, 37200 Tours, France; emilie.allard@univ-tours.fr

**Keywords:** drug delivery, antibody–drug conjugate (ADC), fragment–drug conjugate (FDC), molecular engineering, conjugation motif, bioconjugation, cancer

## Abstract

Antibody–drug conjugates (ADCs) derived from a full immunoglobulin-G (IgG) are associated with suboptimal solid-tumor penetration and Fc-mediated toxicities. Antibody fragment–drug conjugates (FDCs) could be an alternative. Nevertheless, innovative solutions are needed to implant cysteines as conjugation sites in the single-chain fragment variable (scFv) format, which is the backbone from which many other antibody formats are built. In addition, the bioconjugation site has the utmost importance to optimize the safety and efficacy of bioconjugates. Our previous intra-tag cysteine (ITC) strategy consisted of introducing a bioconjugation motif at the C-terminal position of the 4D5.2 scFv, but this motif was subjected to proteolysis when the scFv was produced in CHO cells. Considering these data, using three intra-domain cysteine (IDC) strategies, several parameters were studied to assess the impact of different locations of a site-specific bioconjugation motif in the variable domains of an anti-HER2 scFv. In comparison to the ITC strategy, our new IDC strategy allowed us to identify new fragment–drug conjugates (FDCs) devoid of proteolysis and exhibiting enhanced stability profiles, better affinity, and better ability to kill selectively HER2-positive SK-BR-3 cells in vitro at picomolar concentrations. Thus, this work represents an important optimization step in the design of more complex and effective conjugates.

## 1. Introduction

Antibody–drug conjugates (ADCs) combine a highly potent cytotoxic agent (drug or payload) conjugated through a suitably constructed linker onto a monoclonal antibody (mAb) directed to a tumor-selective antigen [1,2,3,4]. The aims of this approach are to reduce systemic toxicity while enhancing antitumor efficacy. There are currently 14 ADCs approved worldwide that are successfully implemented in clinical strategies [1], while more than 200 clinical trials involving ADCs are actually either recruiting or active [5].

Many of these ADCs are generated using a stochastic bioconjugation process. Site-specific conjugation has been described to improve ADC therapeutic index in comparison to classical random methods [6]. This attractive strategy led to the recent approvals by the Food and Drug Administration of Enhertu^®^ and Trodelvy^®^ (in December 2019 and April 2020, respectively). Despite their recent keen interest, one of the major drawbacks associated with ADCs targeting solid tumors is their insufficient activity at the maximum tolerated dose (MTD) upon repeated doses, leading to many of them being discontinued after failing to progress beyond Phase II. This suggests there are still unmet parameters needing to be optimized in order to reach further translational successes [5,7]. To explain this finding, the efficacy of ADCs based on a full immunoglobulin-G (IgG) format is limited by their size (150 kDa), associated with suboptimal tumor penetration and uptake [8,9]. Furthermore, their Fc portion is considered to mediate off-target toxicity [10,11,12]. Indeed, the long half-life of ADCs [13] due to the neonatal Fc receptor (FcRn) is increasing normal tissue exposure, while Fc-gamma receptors (FcγR) cross-react with endothelial cells and the immune system.

To overcome these drawbacks, several smaller formats of drug conjugates [10,14] have been explored, including peptides [15], single-domain antibody fragments (sdAb or VHH) [16], single-chain fragment variables (scFvs) [17,18], antigen-binding fragment (Fabs) [19] or minibodies (small immunoprotein as scFvs dimerized using a CHε4 domain) [20,21]. Surprisingly, among them, only a few examples of efficiently vectorized drugs with smaller antibody formats have been reported, exhibiting subnanomolar activity with a drug-to-antibody ratio (DAR) between 1 and 2 [19,22,23]. As part of this strategy, we recently described the site-specific conjugation of one monomethyl auristatin F (MMAF) at the C-terminal position onto an engineered anti-HER2 scFv of the trastuzumab antibody (4D5.2), generating an antibody fragment–drug conjugate (FDC) with a DAR of 1 [18,19]. 4D5.2 was produced in the bacteria *Escherichia coli*. A bioconjugation motif including two cysteines (Cys-Gly-Cys) was incorporated at the beginning of the scFv hexahistidine tag, in order to allow controlled bioconjugation of our non-cleavable heterobifunctional linker-MMAF **1** including a diphenylthiomaleimide (DTM) and MMAF [18,24,25,26]. Satisfyingly, our FDC conserved its affinity to HER2 and was able to kill in vitro HER2-positive SK-BR-3 cells at subnanomolar concentrations (EC_50_ of 0.32 nM), while no effect was observed on HER2-negative MCF-7 cells.

However, 4D5.2 was obtained with a relatively low yield classically associated with production in bacteria. Moreover, the intra-tag cysteine (ITC) implantation strategy has shown an important limitation because the tag can be proteolyzed with the loss of the bioconjugation motif. Indeed, when produced in the eukaryotic system (CHO), the scFv 4D5.2 showed a loss of the tag for at least 6% of the fragments produced, characterized by instability over time when stored at 4 °C in PBS. The presence of proteolysis can be a real brake on the future development of biopharmaceuticals. We hypothesize that this phenomenon of proteolysis is due to the length and nature (including cysteines) of the tag, but also to the nature of the amino acid residues present on the surface of the variable domains. Interestingly, the scFv fragment derived from trastuzumab is naturally recognized by protein L; therefore, there is no need to use a tag to purify or detect it [27]. In addition, the bioconjugation site has been described to have the utmost importance to optimize safety, stability, pharmacokinetics, and the therapeutic index of bioconjugates [28,29,30].

**Figure 1:**
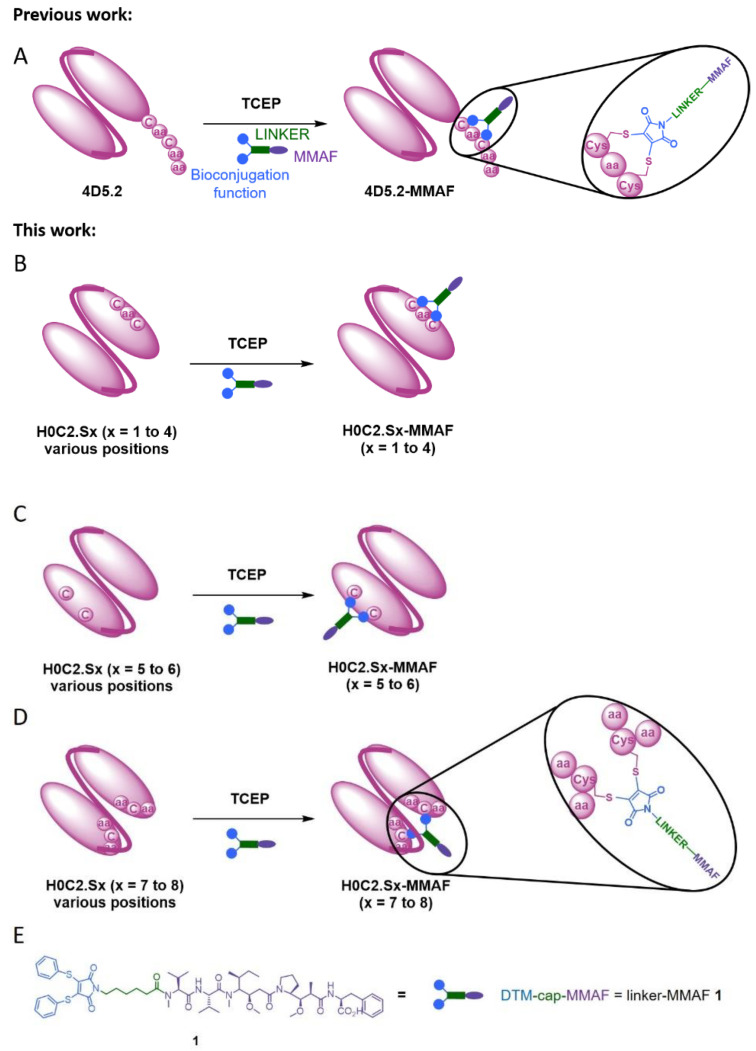
(**A**) Intra-tag cysteine (ITC) strategy: site-specific antibody fragment–drug conjugate (FDC) 4D5.2-MMAF with a drug-to-antibody ratio (DAR) of 1, resulting from the reduction of the single disulfide bridge of 4D5.2 at the C-terminal (and the reduction of 4D5.2 dimer form) with TCEP and conjugation of linker-MMAF **1**, including monomethyl auristatin F (MMAF). (**B**) Linear intra-domain cysteine (IDC) strategy: site-specific FDC H0C2.Sx-MMAF (x = 1 to 4) with a DAR of 1, resulting from the reduction of a single supplementary intra-domain disulfide bridge (the two cysteines are separated only by one amino acid) of H0C2.Sx (and the reduction of H0C2-Sx dimer form) with TCEP and conjugation of linker-MMAF **1**. (**C**) Conformational IDC strategy: site-specific FDC H0C2.Sx-MMAF (x = 5 to 6) with a DAR of 1, resulting from the reduction of the single intra-domain disulfide bridge (the two cysteines are separated by several amino acids on the same variable domain) of H0C2.Sx (and the reduction of H0C2.Sx dimer form) with TCEP and conjugation of linker-MMAF **1**. (**D**) Conformational IDC strategy: site-specific FDC H0C2.Sx-MMAF (x = 7 to 8) with a DAR of 1, resulting from the reduction of the single inter-domain disulfide bridge (the two cysteines are each on a different variable domain) of H0C2.Sx (and the reduction of H0C2-Sx dimer form) with TCEP and conjugation of linker-MMAF **1**. (**E**) Chemical structure of non-cleavable linker-MMAF **1** [18].

In this context, herein, we compared three intra-domain cysteine (IDC) strategies for the incorporation of a cysteine pair directly in the VH and VL domains of the scFv (Figure 1). For this purpose, eight original engineered anti-HER2 scFvs were produced in CHO cells (H0C2.Sx, x = 1 to 8). We assessed the impact of the location of mutation sites on the stability and production yield of each clone and the capacity of each position to allow the site-specific conjugation of one MMAF through our non-cleavable heterobifunctional linker-MMAF **1** (Figure 1). Four clones (H0C2.Sx, x = 3 to 6) were able to afford their respective conjugates H0C2.Sx-MMAF (x = 3 to 6) with an average DAR as close as possible to 1.0 (at least >0.7). We measured the affinity of these four clones to HER2 and their stability, in both their native and conjugated forms. After internalization in HER2-positive cells, the four FDCs H0C2.Sx-MMAF (x = 3 to 6) with a non-cleavable linker are likely to be degraded by lysosomal proteases to release an MMAF metabolite able to kill cancer cells [1,2]. Therefore, we evaluated in vitro their cytotoxicity (vs. 4D5.2-MMAF) on two human breast cancer cell lines: SK-BR-3 (HER2 high expression) and MDA-MB-231 (HER2 low expression).

## 2. Materials and Methods

### 2.1. Protein Expression and Purification

The H0C2 scFv fragment resulted from the association of the heavy and light variable domains of an antibody via the (Gly_4_Ser)_3_ peptide link (without peptide flag in the C-terminal portion). The pcDNA3.4 plasmid was used in the expression of all scFv constructs.

The nucleotide sequences of scFv H0C2 were designed with an optimized codon from *Cricetulus griseus.* The gene was then synthesized and cloned into the pcDNA3.4 plasmid by GeneArt (Thermo Fisher Scientific, Waltham, MA, USA). For the generation of plasmid pcDNA3.4-H0C2.Sx, mutations were introduced by the golden gate technique. Thus, the gene and the plasmid were amplified by PCR using several primers, which allowed the introduction of mutations, as well as the recognition sequence of the Type IIS restriction enzyme. For digestion and ligation, BsaI-HF^®^v2 was used (New England Biolabs). Subsequently, TG1 chemically competent bacteria were transformed with the neo-formed plasmids. All constructs were sequenced and thus confirmed.

Thus, scFv H0C2.Sx (x = 1 to 8) were produced using the ExpiCHO-S cell line (ThermoFisher). Briefly, on the day prior to transfection (Day-1), ExpiCHO-S cells were split to a final density of 4 × 10^6^ viable cells/mL and incubated overnight at 37 °C and 8% CO_2_ under shaking. On the next day (Day 0), cell culture was diluted to 6 × 10^6^ cells/mL and transfected by 0.8 μg/mL of plasmid encoding H0C2 (.Sx), previously mixed to ExpiFectamine CHO reagent. On the day after transfection, the max titer protocol was performed. ExpiFectamine CHO Enhancer and ExpiCHO Feed were added, and cells were incubated at 32 °C with 5% CO_2_ under shaking. On Day 5 post-transfection, a second volume of ExpiCHO Feed was added to the flask. After ten days post-transfection, the supernatant was harvested and purified with an Akta purifier using a HiScreen™ Capto™ L column (Cytiva Europe GmbH, Velizy-Villacoublay, France, 17-5478-14). scFv was eluted by a linear pH gradient in 0.1 M glycine buffer running from pH 6 to pH 2, and the buffer was removed by a desalting column. Antibody concentration was determined with a UV detector at 280 nm. ScFvs molecular mass and molar extinction coefficient data were all generated by the Protparam tool from http://web.expasy.org/protparam/ accessed on 24 May 2019.

### 2.2. Biochemical Characterization and scFv Integrity Analysis

The size and integrity of all purified scFvs were assessed by sodium dodecyl sulfate-polyacrylamide gel electrophoresis (SDS-PAGE) on homogeneous 12% polyacrylamide gel, under denaturation and reducing or non-reducing conditions. Purified scFv samples were all loaded at 1 µg for Coomassie Blue staining (0.1% Coomassie Brilliant Blue R-250, 30% methanol, and 10% glacial acetic acid).

The purified scFv preparations were resolved by size-exclusion chromatography (SEC) on a Superdex 75 10/300 GL column (molecular mass range 3000–70,000) (GE Healthcare Life Sciences, 17-5174-01) with an Äkta purifier. The column was loaded with 20 µg of each scFv construct. Proteins were eluted with PBS at a rate of 0.5 mL/min and detected with a UV detector at 280 nm.

### 2.3. Determination of Thermal Unfolding

Differential scanning fluorimetry experiments were performed on a nanoDSF device (Prometheus NT.48, NanoTemper, München, Germany). All samples were used to a final concentration of 10 µM and loaded into high-sensitivity capillaries. The protein unfolding process was subjected to a thermal ramp (20–95 °C, 1 °C/min). Data analysis was performed using the Prometheus PR ThermControl software. The Tm value was determined by fitting the tryptophan 350/330 nm fluorescence emission ratio using a polynomial function in which the maximum slope is indicated by the peak of its first derivative.

### 2.4. Affinity Analysis by Microscale Thermophoresis

The antigen labeling was carried out according to the instructions in the His-Tag labeling kit Red-tris-NTA (Nanotemper, München, Germany).

Her2-His (Her2/ERBB2 Protein, Human, Recombinant (ECD, His Tag), sinoBiological) was diluted to 200 nM in PBST buffer (137 mM NaCl, 2.5 mM KCl, 10 mM Na_2_HPO_4_, 2 mM KH_2_PO_4_, pH 7.4, 0.05% Tween-20). Tris-NTA dyes were diluted in PBST buffer to a final concentration of 100 nM. A 100 µL volume of protein was then mixed with 100 µL of dye, and the reaction mixtures were incubated for 30 min at room temperature in the dark and then centrifuged for 10 min at 10,000× *g*. The labeling was verified by following the instructions of the pretest of the MO.Control software (Nanotemper, München, Germany).

For the MST binding experiment, the concentration of fragments was diluted to 2 µM in PBS. This solution was used for a 1:1 serial dilution using 16 dilution steps, with a final volume of 6 µL for each point of the dilution series. Afterwards, 6 µL of HER2-Dye was added to all steps of the dilution series, giving a final ligand concentration of 5 nM. The reaction was incubated for 30 min at room temperature and loaded into Monolith NT.115 MST Premium Capillaries. The MST experiment was carried out using 100% LED power and medium MST power for the NT.115 RED instrument (Nanotemper, München, Germany).

### 2.5. Synthesis and Mass Spectrometry on Linker-MMAF 1 and Its Precursors

Synthesis of linker-MMAF **1** was performed according to our previously reported procedure [18]. High-resolution accurate mass measurements (HRAMs) were performed in positive mode with an electrospray ionization (ESI) source on a UHR Q-TOF mass spectrometer (Bruker, Bremen, Germany) with an accuracy tolerance of 2 ppm by the “Fédération de Recherche” ICOA/CBM (FR2708) platform.

### 2.6. Bioconjugation

To scFv H0C2.Sx (x = 0 to 8) (500 μL, 0.12 mg/mL) in BBS (pH 8.0, 1 mM EDTA, 25 mM NaCl) was added TCEP (1 mM, 12 eq.), and the reaction was incubated at 37 °C for 75 min. Linker-MMAF **1** was added as a solution in DMSO (1 mM, 16 eq.), and the reaction was incubated at 4 °C for 16 h under stirring (600 rpm). Crude mixtures were then purified by the Vivaspin centrifugal concentrator (10 kDa MWCO, Sartorius, GE Healthcare, Tremblay-en-France, France) by three cycles (10,000 rpm, 3 min, 4 °C, each) against phosphate-buffered saline (PBS) pH 7.4 and filtered on 0.22 µm membranes (Millex, Sigma Aldrich; Saint-Quentin-Fallavier, France), to afford the desired conjugates H0C2.Sx-MMAF (x = 0 to 8). The protein concentration of purified FDCs was assessed by UV absorption at 280 nm (Nanodrop, Fisher Scientific SAS, Illkirch, France).

### 2.7. Mass Spectrometry on Conjugates

Mass spectrometric analyses of AFCs were performed on a Bruker maXis mass spectrometer coupled to a Dionex Ultimate 3000 RSLC system (Dionex, Germering, Germany) by the “Fédération de Recherche” ICOA/CBM (FR2708) platform. Prior to mass spectrometry (MS) analysis, samples (ca. 1 µg) were desalted on a MassPREP desalting cartridge (2.1 × 10 mm) (Waters, Saint-Quentin-en-Yvelines, France) heated at 80 °C using 0.1% formic acid as solvent A and 0.1% formic acid in acetonitrile as solvent B at 500 µL/min. After 1 min, a linear gradient from 5 to 90% B in 1.5 min was applied; the first 1.5 min were diverted to waste. MS data were acquired in positive mode with an ESI source over the *m*/*z* range from 900 up to 5000 at 1 Hz and processed using DataAnalysis 4.4 SR1 software from Bruker (Bremen, Germany) and the MaxEnt algorithm for spectral deconvolution. Deconvolution was carried out in the range 20–60 kDa. The average drug-to-antibody ratio (*DAR_average_*) was calculated according to a previously reported method. Briefly, the percentage abundance of each *DARi* species represents the relative distribution of each particular drug-loaded FDC species, as monomer and dimer. The *DAR_average_* was then calculated using the percentage peak areas combined with their respective drug load numbers, according to the corresponding formula:

$$DAR_{average}=0\times DAR_{0}+1\times DAR_{1}+2\times DAR_{2}$$

### 2.8. Cell Viability

*Cell culture.* Human breast carcinoma cells MDA-MB-231 were obtained from the European Collection of Authenticated Cell Cultures (ECACC, Salisbury, UK). The cells were grown at 37 °C/5% CO_2_ in Dulbecco’s Modified Eagle Medium (DMEM) with glucose and l-glutamine containing 10% fetal bovine serum (FBS, Thermo Fisher Scientific, Waltham, MA, USA) and 1% penicillin–streptomycin solution (10,000 U/mL, Gibco^®^) and 1% of nonessential amino acid 1X (HyClone Laboratories, Logan, UT, USA). SK-BR-3 cells were obtained from Cell Lines Service (CLS Eppelheim, Eppelheim, Germany). SK-BR-3 cells were maintained in DMEM supplemented with 10% FBS and 1% penicillin–streptomycin solution, in a humidified atmosphere with 5% CO_2_ at 37 °C.

*Cytotoxicity assay.* SK-BR-3 and MDA-MB-231 cells were first plated at a density of 6 × 10^3^ and 3 × 10^3^ cells/mL in 96-well plates for 24 h and then treated with increasing concentrations of various preparations: H0C2.S3-MMAF, H0C2.S4-MMAF, H0C2.S5-MMAF, H0C2.S6-MMAF, 4D5.2-MMAF, MMAF.

The samples were diluted in complete culture medium to obtain concentration from 100 to 0.001 nM. H_2_O_2_ solution at 20 mM and culture medium alone were tested as positive and negative controls. The cells were incubated with 100 µL of each solution at 37 °C/5% CO_2_ for 5 days. Cell viability was then determined using the MTT reagent (Sigma Aldrich; Saint-Quentin-Fallavier, France). Briefly, 10 µL of MTT solution at 5 mg/mL was added to each well, and the plates were incubated at 37 °C for 4 h. The medium was removed, and 200 µL of dimethyl sulfoxide (DMSO) was added to each well and mixed thoroughly to completely dissolve the dark blue crystals. The optical density values were measured at 550 nm using an absorbance microplate reader (Bio-Tek^®^ instruments, Inc., Winooski, VT, USA). The 50% inhibitory concentration (IC_50_) was determined as the MMAF (or equivalent) concentration to induce a 50% reduction in cell viability. Three independent repetition experiments were conducted, each with at least 4 repeated samples.

## 3. Results

### 3.1. Design

With the previous intra-tag cysteine (ITC) strategy, a “CGC” motif was used to allow the bioconjugation of a single linker-MMAF **1** onto the C-terminal position of scFv 4D5.2 [18]. For the new intra-domain cysteine (IDC) strategy, in order to graft the same linker-MMAF **1** directly onto the surface of the scFv, it was important to identify two amino acid residues close in space in order to mimic the “CGC” pattern. In addition, to preserve the integrity of the variable domains and the scFv as much as possible, it was necessary to exclude all the residues not exposed on the surface of the variable domains, as well as those exposed but (i) belonging to the CDRs and, more broadly, to the paratope of the antibody or (ii) at the interface of the VH and VL domains. The positions of amino acid residues in the antibody conformation, especially in the paratope, were identified using the structure of the extracellular region of HER2 in complex with the herceptin Fab (1N8Z) [31]. Side chain solvent accessibility was checked using the website “AAAAA, AHo’s Amazing Atlas of Antibody Anatomy” at http://www.bioc.uzh.ch/antibody, accessed on 18 April 2018 [32].

**Figure 2:**
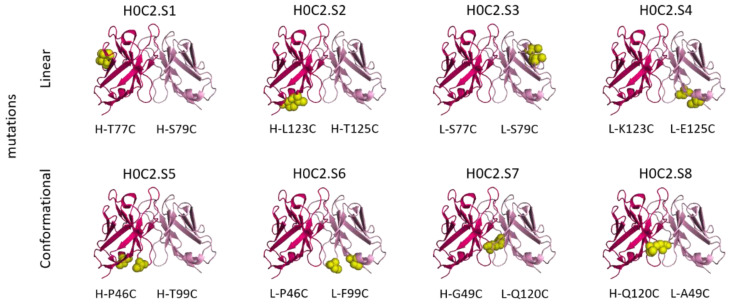
Cysteine positions for the eight different antibody fragments H0C2.Sx (x = 0 to 8) used for the bioconjugation of linker-MMAF **1**. We used the 1N8Z.pdb file, with the VH in hot pink, the VL in pink, and the cysteines in yellow.

Thus, six original double mutations were identified: four according to a linear CxC motif (where the two cysteines are only separated by an amino acid residue) and two according to a conformational motif (e.g., the two cysteines are distant in the amino acid sequence, but nearby in space according to the scFv conformation). The two (already described) cysteine mutations used in the disulfide-stabilized Fv fragments (dsFvs) have also been included, either H-G49C and L-Q120C or H-Q120C and L-A49C [33]. It should be noted that, among the eight proposed scFv H0C2.Sx (x = 0 to 8), mutations are on an equivalent position on the VH or the VL (S1 vs. S3, S2 vs. S4, S5 vs. S6, and S7 vs. S8) (Figure 2).

### 3.2. Production

The scFv fragment H0C2 derived from trastuzumab, used as reference, consists only of the variable domains VH and VL associated with each other by a flexible peptide link in the VH-VL direction. Because this fragment is naturally recognized by the protein L, which allows its rapid and specific purification [27], no flag peptide was used for purification. This also had the advantage of avoiding proteolysis issues.

In H0C2, there is no accessible cysteine available for conjugation. Therefore, for each H0C2.Sx construct, two residues at key positions were mutated to cysteine in order to have a linear or conformational “C.C” motif (Figure 1 and Figure 2). The mutation of two residues to cysteine at any location did not cause any issues during either production or purification. All the fragments could be produced and obtained pure at a concentration of 20 μM (approximately 500 μg/L or 1 of OD at 280 nm). In opposition to 4D5.2, they were stable when stored at 4 °C in PBS or BBS over time.

**Figure 3:**
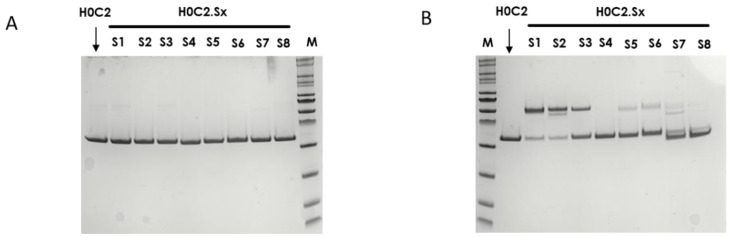
SDS-PAGE analysis to assess the integrity of fragments H0C2 (without intra-domain cysteines (IDC)) and H0C2.Sx (with IDC): (**A**) Denaturating and reducting conditions; (**B**) Denaturating and non-reducting conditions.

In reduced condition, analysis on SDS-PAGE demonstrated that all the fragments migrated according to their expected size without any visible difference (Figure 3A). No proteolysis was observed, even at the level of the conjugation motif. To assess the influence of cysteines on the conformation of the scFvs and their propensity to dimerize, an analysis by SDS-PAGE in non-reducing conditions was performed (Figure 3B).

The proportion between monomer and dimer differs among H0C2 and the eight scFv H0C2.Sx. We observed either only monomer (H0C2), a majority of monomer (H0C2.S4 to H0C2.S8), a balance between monomer and dimer (H0C2.S3), or a majority of dimer (H0C2.S1 and S2). The native fragments were analyzed by mass spectrometry (Figures S1 and S2, Supplementary Materials). The expected molecular weight (in monomer and/or dimer forms) were clearly identified on the chromatograms, accompanied by several peaks corresponding to classical post-translational modifications (e.g., oxidations). These molecular weights corresponded to those of the fragments with the supernumerary cysteines. The disulfide bridges are present either in a single variable domain, or between two variable domains of the same fragment (VH-VL) for a monomer, or between two variable domains of different fragments (VH-VH or VL-VL) for a dimer.

### 3.3. Bioconjugation

The development of the bioconjugation process is a crucial step in the IDC strategy, in order to highlight the discrepancy in bioconjugation behavior between each scFv, to obtain the corresponding FDCs. First, on scFv 4D5.2, from our ITC strategy, compared to our previous publication [18], we optimized the quantities of TCEP reducer (12 eq.) and linker-MMAF **1** (16 eq.) to obtain the FDC 4D5.2-MMAF with an average DAR close to 1 (DAR = 0.84). These are the conditions that will be used then, without modification or optimization, for the bioconjugation of the different scFvs H0C2.Sx (x = 0 to 8), in order to obtain the FDCs H0C2.Sx-MMAF (x = 0 to 8). We used mass spectrometry analysis to identify the efficiency of bioconjugation (Figure S1, Supplementary Materials), which is assessed by observing the different proportions of native fragments (DAR 0) and conjugated fragments (DAR 1, with sometimes a small amount of DAR 2). Bioconjugation is considered satisfactory when the average DAR is as close to 1.0 as possible, there are no or few unconjugated scFvs of DAR = 0, in monomer or dimer form, and the possible presence of DAR = 2 is minimal (Table 1).

### 3.4. Mass Spectrometry Analysis

**Figure 4:**
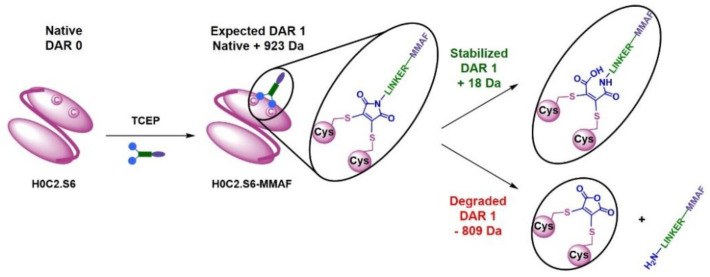
Explanation of analysis of the conjugated fragments H0C2.S6-MMAF. Black species: expected mass increment (+923 Da for DAR 1). Green species: resulted from the stabilization of maleimide(s) into maleic amide(s) by hydrolysis (+18 Da mass increment from a DAR 1). Red species: resulted from the deconjugation (loss) of an aminocaproic-MMAF on a DAR 1 and transformation of maleimide into maleic anhydride (−809 Da loss of mass from DAR 1, also corresponding to a +114 Da mass increment from a native DAR 0).

All clones were analyzed by mass spectrometry (MS), in their native and conjugated forms (Figure S1, Supplementary Materials). The bioconjugation results made it possible to classify the fragments into three categories (Figure 4 and Table 1). The first, represented by H0C2.S1 and H0C2.S2, showed an absence of conjugate but a high proportion of native dimer. The reduction was therefore not sufficient to reduce the inter-scFv disulfide bridges (VH-VH disulfide bonds, between two VH domains of two scFvs).

The second category corresponds to H0C2.S7 and H0C2.S8. Bioconjugation was weak to non-existent, but without dimeric forms, in contrast to the first category.

The third category is represented by the scFvs H0C2.S3 to H0C2.S6 whose bioconjugation is satisfactory, with the corresponding FDCs exhibiting an average DAR > 0.7. These results are similar to what we previously obtained with the 4D5.2 fragment. Increment analysis showed that for DAR 1, the increment is +923 Da as expected. For H0C2.S3 to H0C2.S6, the proportion of the different species after bioconjugation would seem to demonstrate that the average DAR obtained would depend on three main factors: (i) the presence of non-reduced dimer, (ii) the stabilization of the maleimide, and/or (iii) destabilization of maleimide, the latter two depending on the protein environment nearby the conjugated cysteines. First, after bioconjugation, 20% dimer remains for H0C2.S3 and 14% for H0C2.S4. Secondly, it can be observed in H0C2.S3 that 11% of unconjugated monomer remains, and 4% for H0C2.S4. Thirdly, for H0C2.S6, three things are observed: (i) there is no more dimer, (ii) there is only 68% DAR 1 monomer, entirely stabilized by the hydrolysis of maleimide into maleic amide (increment +18 Da), and (iii) there is 28% of degraded DAR 1, corresponding to the loss of the linker after the transformation of maleimide into maleic acid (−809 Da loss of mass from DAR 1). The conjugate H0C2.S5-MMAF was almost perfect, homogeneous with an average DAR (1.08) slightly greater than 1.0 because of a minor presence of the DAR 2 fragment.

**Table 1:** Analysis of the conjugated fragments 4D5.2-MMAF and H0C2.Sx-MMAF (x = 0 to 8) by MS according to their oligomerization state and calculated average DAR. Black species: expected mass increment (+923 Da for DAR 1 or +1846 Da for DAR 2). Green species: resulted from the stabilization of maleimide(s) into maleic amide(s) by hydrolysis (+18 Da mass increment from a DAR 1 or +36 Da mass increment from a DAR 2). Red species: resulted from the deconjugation (loss) of an aminocaproic-MMAF on a DAR 1 and transformation of maleimide into maleic anhydride (−809 Da loss of mass from DAR 1, also corresponding to a +114 Da mass increment from a native DAR 0).

Conjugated Fragments	% of Monomer			% of Dimer			Average DAR
	DAR 0	DAR 1	DAR 2	DAR 0/0	DAR 0/1	DAR 1/1	
H0C2	100						0
4D5.2	28	60	12				0.84
HOC2.S1	10			90			0
HOC2.S2	5			95			0
HOC2.S3	11	65	4	20			0.73
HOC2.S4	4	72	10	14			0.92
HOC2.S5		90	9	1			1.08
HOC2.S6	28	68	4				0.76
HOC2.S7	40	49	2	4	4	1	0.56
HOC2.S8	100						0

### 3.5. SEC Analysis of scFvs and FDCs

**Figure 5:**
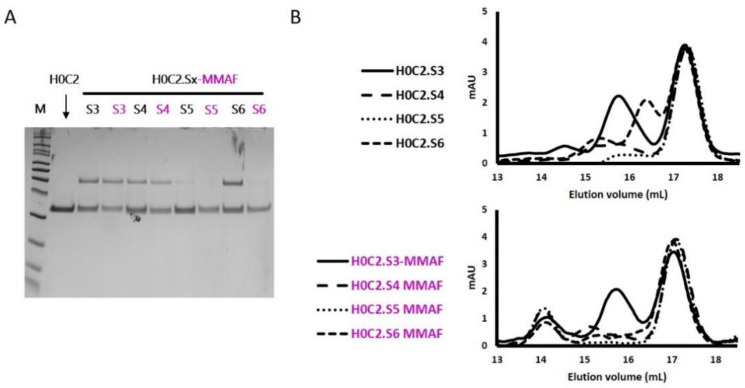
(**A**) Denaturating and non-reducting SDS-page analysis of fragments H0C2.Sx (x = 3 to 6), in native (black) and conjugated (purple) forms. (**B**) Analysis of native fragments H0C2.Sx (x = 3 to 6) and conjugated fragments H0C2.Sx-MMAF (x = 3 to 6), by size-exclusion chromatography (SEC).

The four fragments H0C2.S3 to H0C2.S6, and their corresponding FDCs H0C2.S3-MMAF to H0C2.S6-MMAF, were analyzed by SDS-PAGE and size-exclusion chromatography (SEC) (Figure 5). These techniques confirmed the results obtained by mass spectrometry, in particular the presence of an important amount (20%) of dimer in FDC H0C2.S3-MMAF, whereas it was lower for H0C2.S4-MMAF (14%), or even non-existent for FDC H0C2.S5-MMAF and H0C2.S6-MMAF. SEC analysis confirmed that the monomers were bioconjugated with an elution volume increasing from 17.3 mL to 17.07 mL. It also showed, for the 4 FDCs H0C2.Sx-MMAF (x = S3 to S6), the presence of a new oligomer (about 15%) at an elution volume of 14 mL.

### 3.6. Thermal Analysis of scFvs and FDCs

An interesting point was the study of the thermal stability of the native fragments and especially of the FDCs (Table 2). First, the implantation of the two cysteines did not modify the conformation of the scFv fragments for H0C2.S6 with a melting temperature (Tm) identical to the control fragments (4D5.2 with flag peptide, and H0C2 without). For H0C2.S3 and H0C2.S4, the Tm was slightly lowered.

On the other hand, for H0C2.S5, the Tm was reduced by 9.3 °C. The conjugation of linker-MMAF **1** on the different scFvs led to a slight decrease in Tm (from −1.5 to −2.2 °C), even for 4D5.2. The loss was also greater for FDC H0C2.S5-MMAF (−3.1 °C). Only the FDC H0C2.S4-MMAF showed an identical Tm compared to its native counterpart. Thus, the different FDCs retained the initial conformation of their native scFv precursor, except FDC H0C2.S5-MMAF.

### 3.7. Affinity to HER2 of scFvs and FDCs

Then, before the cytotoxicity evaluation, it was important to check if chemical modifications, following bioconjugation, did not affect the affinity of the fragments for their specific antigen HER2. This exploration was carried out by thermophoresis in soluble condition (Figure S3, Supplementary Materials). The first important information was that the implantation of the cysteines did not modify the affinity of the different native fragments for HER2 (Table 3). Indeed, for 4D5.2 and the scFv H0C2.Sx (x = 3 to 6), the difference in affinity is small in comparison to the reference fragment H0C2, with a K_D_ of less than one log lower. On the other hand, for 4D5.2-MMAF and the FDCs H0C2.Sx-MMAF (x = 3 to 6), the presence of the linker slightly decreased the affinity (K_D_ of approximately one log lower), except for H0C2.S3-MMAF with a K_D_ of two logs lower than H0C2.S3. Nevertheless, the affinity remained high, approximately 10 nM for FDC H0C2.S4-MMAF and H0C2.S6-MMAF or more than 30 nM for H0C2.S3-MMAF and H0C2.S5-MMAF.

### 3.8. Cell Viability

**Figure 6:**
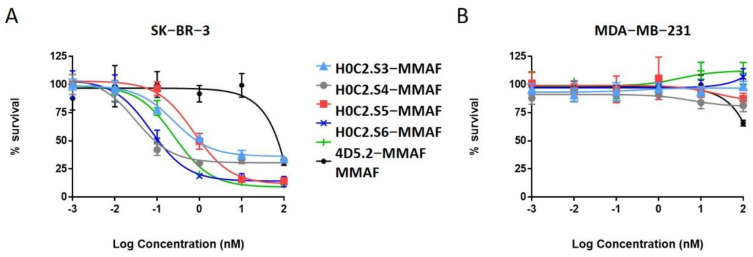
Cytotoxicity data for FDCs 4D5.2−MMAF, H0C2.Sx−MMAF (x = 3 to 6) and unconjugated MMAF (**A**) on the HER2-overexpressing cell line SK−BR−3 and (**B**) on the HER2 low expressing cell line MDA−MB−231.

We evaluated in vitro the cytotoxicity of the best FDCs H0C2.Sx-MMAF (x = 3 to 6) and their corresponding native scFv, compared to our previous FDC 4D5.2-MMAF and scFv 4D5.2, on two human breast cancer cell lines: SK-BR-3 (HER2 high expression) and MDA-MB-231 (HER2 low expression). As expected, no toxicity was observed neither for the native fragments on the two cell lines (Figure S4, Supplementary Materials), nor for the FDCs on the MDA-MB-231 cell line (Figure 6B). On the contrary, all the FDCs were cytotoxic at different concentrations on the SK-BR-3 cell line (Figure 6A and Figure S5, Supplementary Materials). For FDC 4D5.2-MMAF, a similar EC_50_ was found (EC_50_ = 0.261 ± 0.057 nM) in comparison to our previous study (EC_50_ = 0.320 nM) [18]. Compared to this reference, on the one hand, FDC H0C2.S5-MMAF was significantly three times less toxic (EC_50_ = 0.775 ± 0.264 nM, *p* value = 0.0009), while FDC H0C2.S3-MMAF was similar (EC_50_ = 0.301 ± 0.200 nM). On the other hand, two FDCs exhibited higher cytotoxicity: H0C2.S6-MMAF was significantly more than three times more toxic (EC_50_ = 70 ± 11 pM, *p* value = 0.0001), while H0C2.S4-MMAF was significantly eight times more toxic (EC_50_ = 31 ± 5 pM, *p* = 0.0001).

## 4. Discussion

Armed antibodies currently represent sophisticated targeted therapies. Although the ADC concept is quite simple, its implementation is very challenging, and limitations still exist. Improving ADC can still be achieved by several strategies (e.g., linker, payload, release mechanisms), including the optimization of the antibody format used to vectorize the cytotoxic conjugated compound, to reach better tumor penetration and biodistribution profile. As part of this international effort, we recently described a first strategy, the intra-tag cysteine (ITC) strategy, with the site-specific conjugation of one MMAF through our linker-MMAF **1**, onto a bioconjugation motif including two cysteines at the C-terminal position of an anti-HER2 scFv of the humanized antibody trastuzumab (4D5.2), to generate an FDC with a DAR of 1 [18]. The obtained FDC, 4D5.2-MMAF, conserved its affinity to HER2 and was able to selectively kill in vitro HER2-positive SK-BR-3 cells with an EC_50_ of 0.32 nM. However, the ITC implantation strategy was limited by the tag proteolysis associated with the loss of the bioconjugation motif, which constitutes a real brake on the future development of more complex biopharmaceuticals, using the scFv as a building block. Linker-MMAF **1** was first designed to rebridge IgG disulfide bridges, after mild reduction, to lead to an ADC with a DAR 4 [26]. We also demonstrated that linker-MMAF **1** was efficient for the ITC strategy [18]. However, in these previous studies, we did not evaluate the minimum or maximum distance required between the two cysteine side chains to afford a bioconjugation when using linker-MMAF **1**.

Thus, we developed here a second strategy, the intra-domain cysteine (IDC) strategy, where four solutions have been identified to reproduce this “CxC” linear motif (H0C2.S1 to S4). In parallel, the cysteines could also be a “C.C” conformational motif (H0C2.S5 to S8), where the cysteine residues are nearby in space, while distant in the amino acid sequence, according to the three-dimensional structure of the scFv conformation. The different cysteine positions clearly influenced the covalent oligomerization of the scFv fragments. The “linear” mutations were made in a comparable way on the VH and VL. However, the S1 or S2 positions on the VH, respectively, equivalent to S3 or S4 on the VL, led to a different result, in particular with a large majority of dimer for S1 and S2. For S7 and S8, the mutations were made symmetrically on the VH and VL resulting in the formation of an inter-domain disulfide bridge. This was checked on SDS-PAGE by the greater migration of S7 and S8 compared to the other fragments. When the SDS-PAGE analysis was performed under reductive conditions (Figure 3A), all fragments appeared completely reduced, suggesting that the formation of disulfide bridges, intra- or inter-domain, should not be a restriction for bioconjugation. Thus, the eight solutions proposed for the implantation of two cysteines directly in the variable domains were validated by obtaining all the purified fragments. For H0C2.S5, a reduced Tm (by 9.3 °C) was intriguing, especially since H0C2.S6 (similar mutations but on the VL) share the same Tm with reference H0C2. The mutated proline in the PG motif, responsible for the formation of a C-C′ loop between CDR1 and CDR2, certainly has a more crucial role in the VH domain than VL.

The bioconjugation results made it possible to classify the fragments into three categories. In the first category, H0C2.S1 and H0C2.S2 were associated with an absence of conjugation with a high proportion of native dimer. This phenomenon has not been observed with H0C2.S3 and H0C2.S4 (on identical positions), which could be surprising because H0C2.S3 is the equivalent of H0C2.S1 and H0C2.S4 is the equivalent of H0C2.S2. Thus, the phenomenon seems to be linked more to the nature of the domain than to the location of the cysteines (S3 similar to S1 and S4 similar to S2). Probably, the environment of the cysteines implanted in the VL domains would not favor a strong dimerization between two VL domains. This phenomenon is probably related to the nature of H0C2 and could be different with another antibody. It is possible to reduce S1 and S2 but with an important number of equivalents of TCEP (more than 200 eq., data not shown) not compatible for further development in a bioconjugation process.

In the second category, H0C2.S7 and H0C2.S8 were associated with a weak to non-existent bioconjugation. The reason is different from the first category, because there are no dimeric forms. Structurally, the implanted cysteines are less exposed on the surface of the scFv, making them good candidates for an intra-domain disulfide bridge (dsFv) but not for a bioconjugation site. It is also very likely that the cysteine environment is crucial, since H0C2.S7 was weakly bioconjugated with an average DAR of 0.5, unlike H0C2.S8.

In the third category, the best bioconjugations were observed with H0C2.S3 to H0C2.S6, and following our results, it is possible to classify the conjugation sites from least to most favorable: S6 < S3 < S4 < S5. Although it was not the goal of this study, it is noteworthy to add that the average DAR was probably improvable by (i) a slightly better reduction for S4 and S3, to avoid the remnant native dimer, and (ii) by a number of linker equivalent being optimized to avoid a non-conjugating part of the native monomer. DAR 2 fragments were observed on all the conjugable fragments (H0C2.S3 to H0C2.S6). Concerning the stabilization of the conjugated linker by the close protein environment, on the one hand, we noted that only for H0C2.S6-MMAF of DAR 1, an increment of +18 Da was observed on the DAR 1. On the other hand, for the DAR 2, all the linkers were stabilized, and this was visible by an increment of 2 × 18 = 36 Da. Observing DAR 2 was not necessarily an issue, as it was always in minor proportion, and always observed with this bioconjugation technology in previous publications [18,19,26]. Therefore, we decided that only scFv H0C2.Sx (x = 3 to 6) were suitable to continue the rest of the study, where the two implanted cysteines were correctly incorporated to obtain a satisfactory average DAR (>0.7) for the conjugates.

For the four selected conjugates H0C2.Sx-MMAF (x = S3 to S6), SEC analysis showed the presence of a new oligomer (about 15%) at an elution volume of 14 mL, most likely due to aggregation associated with the hydrophobic character of linker-MMAF **1**. Concerning the affinity to HER2, for FDCs 4D5.2-MMAF and the H0C2.Sx-MMAF (x = 3 to 6), the presence of the linker slightly decreased the affinity when compared to their respective native scFv (K_D_ of one log lower), except for H0C2.S3-MMAF when compared to H0C2.S3 (K_D_ of two logs lower). The position of the linker on the side of the fragment, therefore closer to the recognition site of HER2, could be responsible for this greater loss. The positioning of the linker, located opposite the paratope, would seem more favorable, even if the loss of one log, associated with all conjugated fragments, was difficult to explain. Probably, the hydrophobic charge of linker-MMAF **1** changed the environment of the fragment and did not facilitate interaction with a large antigen such as HER2. According to the affinity ranking, the least to the most favorable location of MMAF would be: the flank of VH domain (S3) *<* the bottom of VH domain (S5) *<* the bottom of VL domain (S4, S6, as well as 4D5.2).

No cytotoxic activity was observed with the native scFv, or the untargeted MMAF payload alone (due to a charged carboxylic acid at physiological pH), on both cell lines. Only FDCs H0C2.Sx-MMAF (x = S3 to S6) and 4D5.2-MMAF were cytotoxic on the HER2-positive SK-BR-3 cell line while sparing the HER2-low MDA-MB-231 cell line. This demonstrated that the therapeutic effect of FDCs H0C2.Sx-MMAF (x = S3 to S6) and 4D5.2-MMAF was due to HER2-targeted uptake of the FDCs rather than non-specific protein uptake, and was limited to the HER2-overexpressing cell line. The difference in cytotoxicity between FDCs H0C2.Sx-MMAF (x = S3 to S6) and 4D5.2-MMAF, and their native counterparts used as control, confirmed that cell killing was via payload-mediated cytotoxicity rather than a therapeutic effect from the antibody fragment alone.

The most homogeneous FDC was obtained for H0C2.S5-MMAF (without DAR 0), with the highest average DAR (1.08). However, H0C2.S5-MMAF was the least cytotoxic FDC, probably due to a conformation issue, observable in particular by a strong drop in Tm and a rather lower affinity to HER2. In contrast, with a lower DAR (0.73) and the presence of competitive inhibitor DAR 0 (11% monomer and 20% dimer), with a similar affinity to HER2, FDC H0C2.S3-MMAF exhibited higher cytotoxicity than FDC H0C2.S5-MMAF and similar to reference 4D5.2-MMAF. The only factor that could explain this result and be in favor of FDC H0C2.S3-MMAF would be the position of the linker on the VL. Indeed, the results for FDCs H0C2.S4-MMAF and H0C2.S6-MMAF confirmed this statement: these two conjugated fragments exhibited the best toxicity and was similar to H0C2.S3-MMAF from the grafting of linker-MMAF **1** onto a basal position in the VL domain.

The more homogeneous DAR for H0C2.S4-MMAF, compared to H0C2.S6-MMAF, could explain its better cytotoxicity on SK-BR-3 cell line, reaching quite an impressive EC_50_ of 31 ± 5 pM for an FDC with an average DAR of only 1, equivalent to an EC_50_ observable for an ADC of DAR 4 [26].

## 5. Conclusions and Prospects

We developed eight original scFvs (new IDC strategy), where no flag peptide was needed, and two residues at key positions were mutated to cysteine in order to have a linear or conformational “C.C” bioconjugation motif. Our results showed no proteolysis issue and satisfying production yields in CHO cells for new scFvs H0C2.S1 to H0C2.S8. In addition, we managed to demonstrate that the reaction of linker-MMAF **1** was more or less difficult depending on the location and accessibility of the bioconjugation site onto the scFvs. Indeed, among the eight proposed scFvs, only four scFvs (H0C2.S3 to H0C.S6) displayed excellent bioconjugation ability, leading to the corresponding FDCs H0C2.Sx-MMAF (x = 3 to 6) with an average DAR close to 1.0.

Another objective was to determine if the localization of the linker could affect the efficiency of the FDCs. Two FDCs, H0C2.S4-MMAF and H0C2.S6-MMAF, exhibited a significantly higher EC_50_ than our previous reference FDC 4D5.2-MMAF [18] on the SK-BR-3 cell line (HER2 high expression). The location of linker-MMAF **1** onto a basal position in the VL domain thus seems to be an important criterion to optimize FDCs. More precisely, in the case of H0C2.S4-MMAF, the two cysteines were located on the FR4 of the VL, a sequence that remains relatively constant. It is very likely that the results obtained on the scFv fragment of herceptin can easily be transposed to other antibodies. The characteristics of scFv H0C2.S4 or FDC H0C2.S4-MMAF were relatively similar to those of scFv 4D5.2 or FDC 4D5.2-MMAF, respectively, although H0C2.S4 (native and conjugated forms) exhibited neither proteolysis nor degradation of the conjugated linker. Therefore, the localization of linker-MMAF **1** onto a basal position in the VL domain (IDC strategy) was better than on the tag (ITC strategy) to produce FDCs with optimized properties and increased efficacy.

Finally, the scFv format is associated with a too fast clearance to be effective in vivo. Nevertheless, the scFv is very interesting as the backbone from which many other antibody formats are built. Thus, this original IDC strategy is an innovative tool to create new conjugation sites on more complex antibody formats (e.g., minibody, diabody, scFv-Fc). These larger antibody formats should have better pharmacokinetic properties and should be better suited against solid tumors. We believe that this new advancement in antibody technology could be implemented to produce novel and more effective antibody–drug conjugates as targeted cancer therapies, to explore the full potential of our IDC strategy.

## Figures and Tables

**Table 2:** Thermal stability of the native fragments H0C2, 4D5.2 and H0C2.Sx (x = 3 to 6), and conjugated fragments 4D5.2-MMAF and H0C2.Sx-MMAF (x = 3 to 6). Tm: melting temperature (corresponding to 50% of unfolded protein); ΔTm and ΔTm (C − Ref): variation of Tm in comparison to the reference fragment H0C2; ΔTm (C − N): variation of Tm between a conjugated fragment and its native precursor.

	Native fragments (N)		MMAF Conjugated Fragments (C)		
	Tm	ΔTm (N − Ref)	Tm	ΔTm (C − N)	ΔTm (C − Ref)
H0C2 (Ref)	68.1 ± 0.3				
4D5.2	68.1 ± 0.1	0	66.6 ± 0.1	−1.5	−1.5
H0C2.S3	66.9 ± 0.2	−1.2	65.4 ± 0.2	−1.5	−2.7
H0C2.S4	66.4 ± 0.3	−1.7	66.3 ± 0.2	−0.1	−1.8
H0C2.S5	58.8 ± 0.4	−9.3	55.7 ± 0.4	−3.1	−12.4
H0C2.S6	68.1 ± 0.4	0	65.8 ± 0.1	−2.3	−2.3

**Table 3:** Equilibrium dissociation constant K_D_ measuring the binding affinity of the native fragments H0C2, 4D5.2 and H0C2.Sx (x = 3 to 6) or the conjugated fragments 4D5.2-MMAF and H0C2.Sx-MMAF (x = 3 to 6) towards their specific antigen HER2.

	Native Fragments (N)	MMAF Conjugated Fragments (C)	
	K_D_	K_D_	K_D_ Ratio (C/N)
H0C2 (Ref)	4.77 × 10^−9^		
4D5.2	1.05 × 10^−9^	1.48 × 10^−8^	14
H0C2.S3	6.71 × 10^−10^	6.21 × 10^−8^	93
H0C2.S4	9.39 × 10^−10^	9.86 × 10^−9^	11
H0C2.S5	5.14 × 10^−9^	3.42 × 10^−8^	7
H0C2.S6	8.07 × 10^−10^	1.36 × 10^−8^	17

## Supplementary Materials

The following supporting information can be downloaded at: https://www.mdpi.com/article/10.3390/pharmaceutics14081524/s1, Figure S1: Mass spectrometry analysis of native and conjugated scFv; Figure S2: relative percentage of monomer and dimer in each native scFv; Figure S3: equilibrium dissociation constant K_D_ measurement for native and conjugated scFv; Figure S4: cell viability study for native scFv; Figure S5: statistical significance calculations for cytotoxicity data of conjugates on SK-BR-3 cell line.
