# Supplementary material for: Intra-Domain Cysteines (IDC), a New Strategy for the Development of Original Antibody Fragment–Drug Conjugates (FDCs)
Source: Pharmaceutics. 2022 Jul 22;14(8):1524. doi: 10.3390/pharmaceutics14081524 (PMC9331466; doi:10.3390/pharmaceutics14081524)
Supplement: Supplementary file 1 [file pharmaceutics-14-01524-s001.zip › pharmaceutics-1816993-supplementary.pdf]

SUPPORTING INFORMATION

Intra-domain cysteines (IDC), a new strategy for the development of original antibody fragment-drug conjugates (FDC).

Louis Jolivet,[1,a] Imène Ait Mohamed Amar,[2,a] Catherine Horiot,[1] Fanny Boursin,[1] Cyril Colas,[4,5] Stéphanie Letast,[2] Caroline Denevault-Sabourin,[2] Emilie Allard-Vannier,[3] Nicolas Joubert,[2,*] and Nicolas Aubrey[1]

[1] ISP UMR 1282, INRA, Université de Tours, Team BioMAP, 31 avenue Monge, 37200 Tours, France;
[2] GICC EA7501, Team IMT, Université de Tours, UFR de médecine, Bâtiment Vialle, 10 Boulevard Tonnelé, BP 3223, 37032 Tours cedex 01, France;
[3] NMNS EA 6295, Université de Tours, 31 avenue Monge, 37200 Tours, France;
[4] ICOA UMR 7311, Université d'Orléans, CNRS, rue de Chartres, 45067 Orléans, France;
[5] CBM UPR 4301, CNRS, Université d'Orléans, rue Charles Sadron, 45071 Orléans, France.
[a] These authors contributed equally to this work.
* Correspondence: nicolas.joubert@univ-tours.fr

TABLE OF CONTENT

Mass spectrometry analysis of native and conjugated scFv……………………………………………..…..S2

Relative percentage of monomer and dimer in each native scFv………………………………………...S4

Equilibrium dissociation constant K_D measurement for native and conjugated scFv …….……..……..S5

Cell viability study for native scFv…………………………………………………………………….....S5

Statistical analysis for cytotoxic data.................................……………………………………………….S5

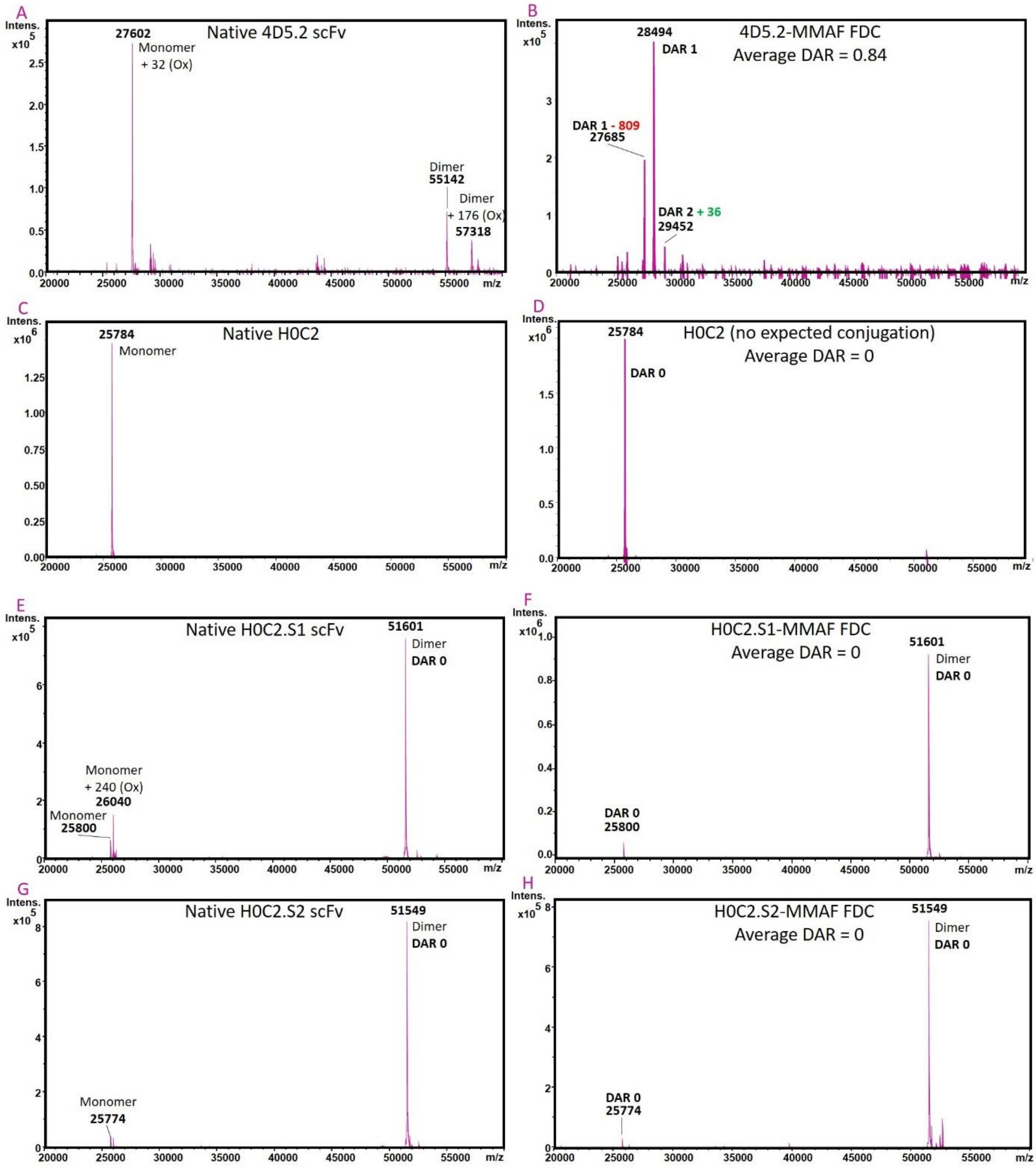

Figure S1. Deconvoluted mass spectra of (A) native scFv 4D5.2, observed as oxidized monomer (MW +32 Da), dimer (MW x2) and oxidized dimer (MW x2 +176 Da). Oxidized species (and dimers when possible) are expected to disappear after mild reduction. (B) FDC 4D5.2-MMAF presenting (i) a +923 Da mass increment (expected +923 Da) corresponding to a DAR 1 monomer with the conjugation of one linker-MMAF **1**, (ii) a second species (degraded DAR 1, DAR 1 - 809 Da) resulting from the deconjugation (loss) of an aminocaproic-MMAF from a DAR 1 and transformation of maleimide into maleic anhydride, and (iii) a third species with a +1846 Da mass increment, corresponding to the conjugation of two linker-MMAF **1**, with both stabilized (opened/hydrolyzed) maleimide (+18 Da x2), leading to a stabilized DAR 2 monomer (MW +1846 Da + 36 Da). (C) native reference scFv H0C2, on which no conjugation is expected. (D) unconjugated H0C2. (E) native scFv H0C2.S1, observed as monomer (MW), oxidized monomer (MW +240 Da) and dimer (MW x2). (F) FDC H0C2.S1-MMAF, observed as DAR 0 monomer (MW) and DAR 0 dimer (MW x2). (G) native scFv H0C2.S2, observed as monomer (MW) and dimer (MW x2). (H) FDC H0C2.S2-MMAF, observed as DAR 0 monomer (MW) and DAR 0 dimer (MW x2).

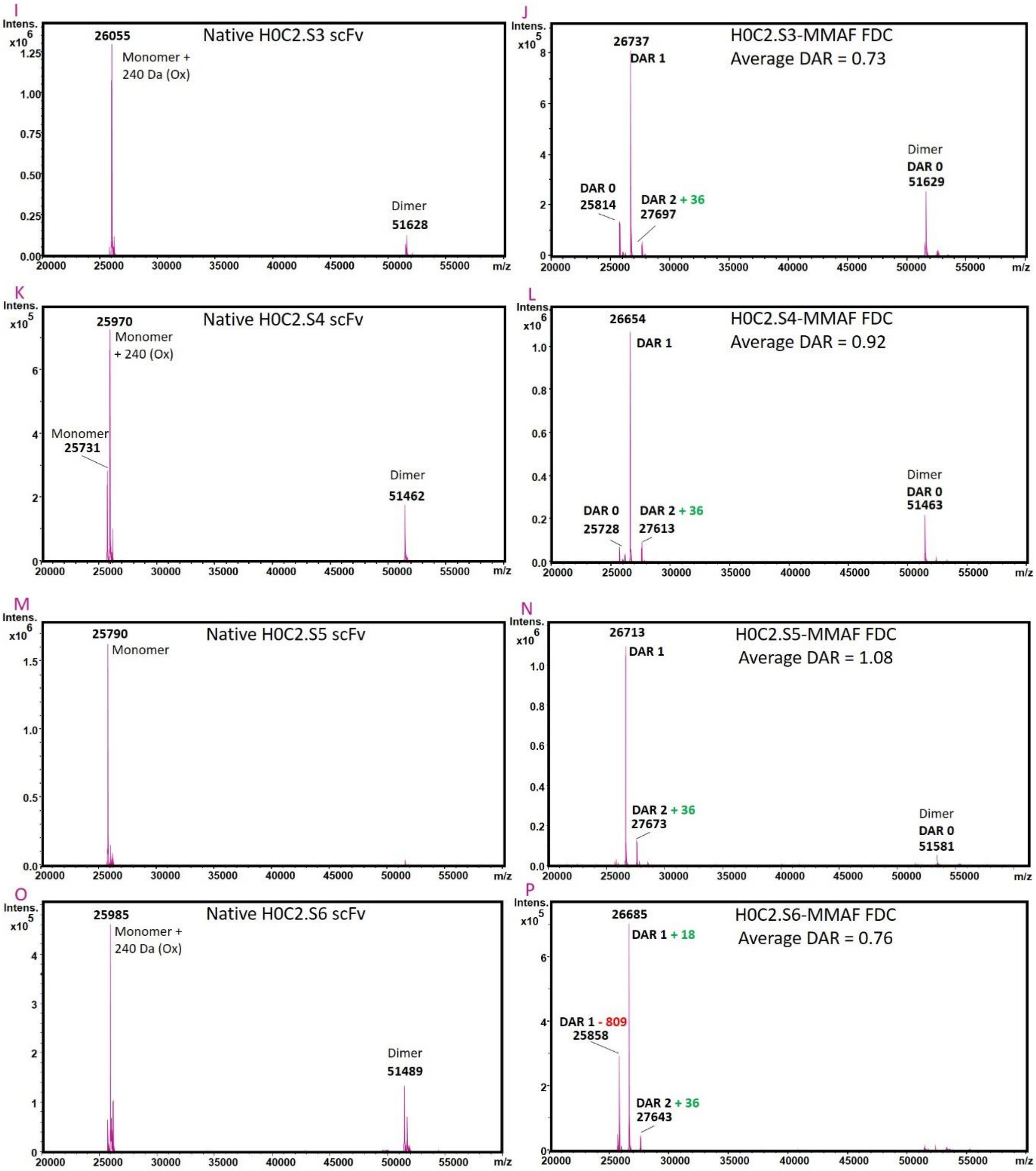

Figure S1 (continued). Deconvoluted mass spectra of (I) native H0C2.S3, observed as oxidized monomer (MW +240 Da) and dimer (MW x2) (J) FDC H0C2.S3-MMAF as four species: DAR 0 monomer (MW), DAR 1 monomer (MW +923 Da), stabilized DAR 2 monomer (MW + 1846 Da + 36 Da) and DAR 0 dimer (MW x2). (K) native scFv H0C2.S4, observed as monomer (MW), oxidized monomer (MW +240 Da) and dimer (MW x2). (L) FDC H0C2.S4-MMAF as four species: DAR 0 monomer (MW), DAR 1 monomer (MW +923 Da), stabilized DAR 2 monomer (MW +1846 Da +36 Da) and DAR 0 dimer (MW x2). (M) native scFv H0C2.S5 as a monomer (N) FDC H0C2.S5-MMAF as three species: DAR 1 monomer (MW +923 Da), stabilized DAR 2 monomer (MW +1846 Da +36 Da) and DAR 0 dimer (MW x2). (O) native scFv H0C2.S6, as oxidized monomer (MW +240 Da) and dimer (MW x2). (P) FDC H0C2.S6-MMAF as three species: degraded DAR 1 monomer (MW +923 Da -809 Da), stabilized DAR 1 monomer (MW +923 Da +18 Da), and stabilized DAR 2 monomer (MW +1846 Da +36 Da).

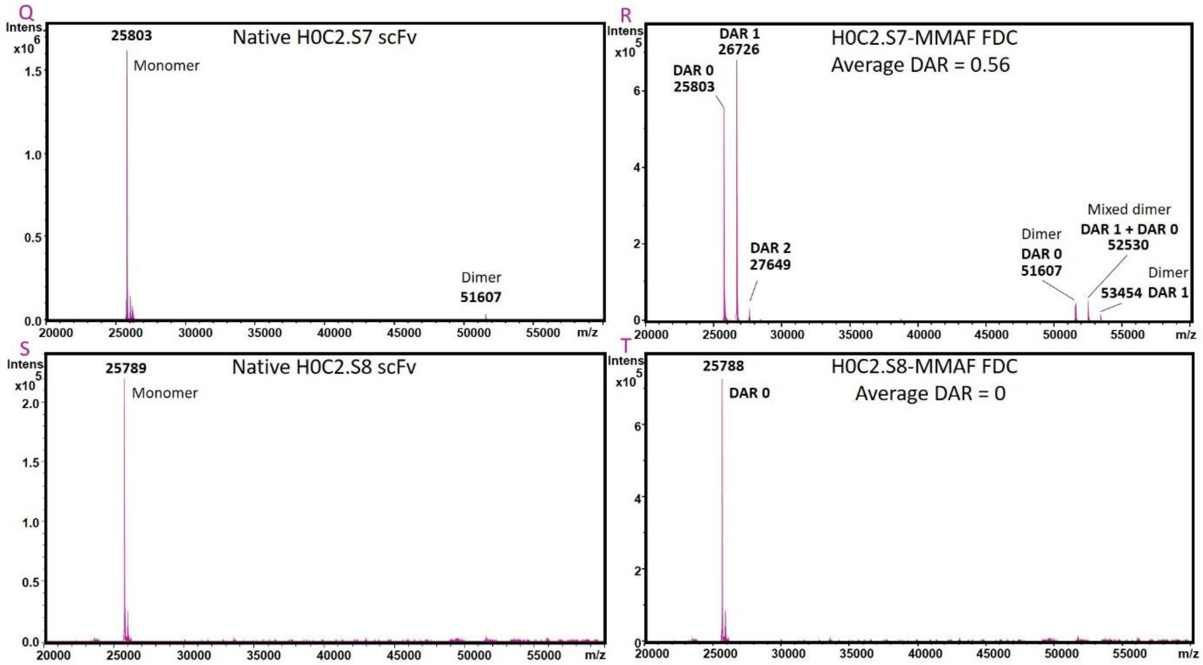

Figure S1 (continued). Deconvoluted mass spectra of (Q) native H0C2.S7, observed as monomer (MW) and dimer (MW x2) (R) FDC H0C2.S7-MMAF as six species: DAR 0 monomer (MW), DAR 1 monomer (MW +923 Da), DAR 2 monomer (MW +1846 Da +36 Da), DAR 0 dimer (MW x2), mixed DAR 0 and DAR 1 dimer (MW x2 +923 Da) and DAR 1 dimer (MW x2 +1846 Da). (S) native scFv H0C2.S8 as monomer (MW). (T) FDC H0C2.S8-MMAF as DAR 0 monomer (MW).

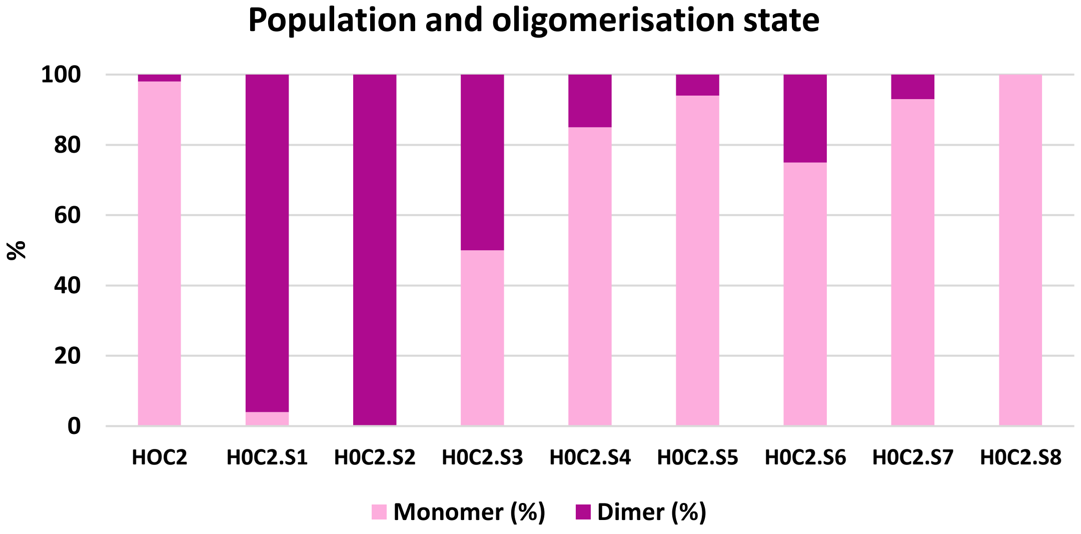

Figure S2. Relative percentage of monomer and dimer in each native scFv.

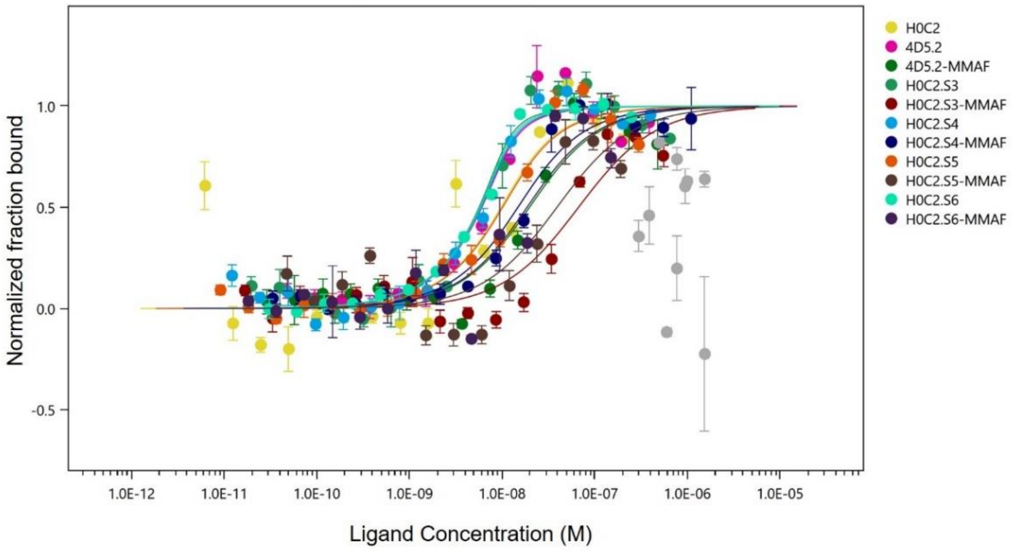

Figure S3. Overview of the equilibrium dissociation constant K_D measurements for the native fragments H0C2, 4D5.2 and H0C2.Sx (x = 3 to 6) or the conjugated fragments 4D5.2-MMAF and H0C2.Sx-MMAF (x = 3 to 6) towards their specific antigen HER2.

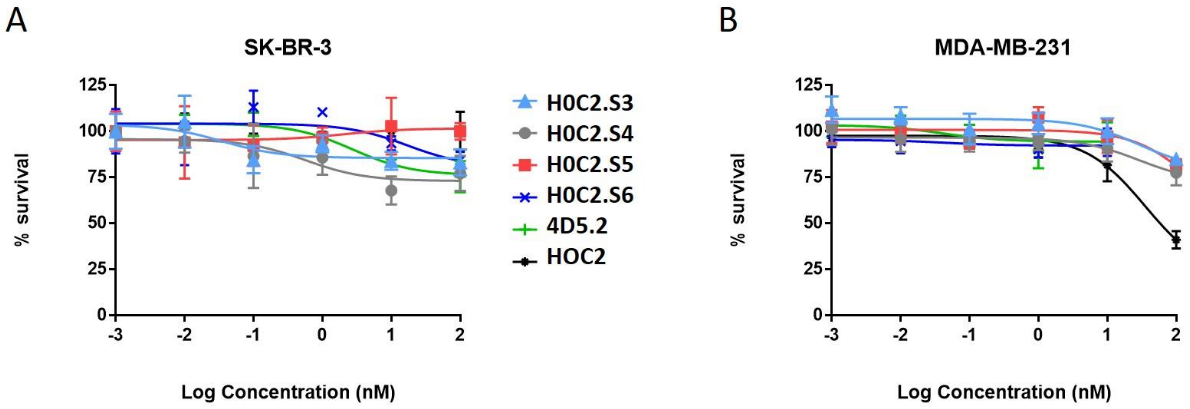

Figure S4. Cytotoxicity data for scFv 4D5.2, H0C2 and H0C2.Sx (x = 3 to 6) (A) on the HER2 overexpressing cell line SK-BR-3 and (B) on the HER2 low expressing cell line MDA-MB-231.

	EC_{50} (nM)	*P* value	T-score	df	Standard error of difference	Significance
4D5.2-MMAF (EC_{50} = 0.261 ± 0.057)	**H0C2.S3-MMAF**					
	0.301 ± 0.200	0.6478	0.4709	10	0.085	Not significant
	**H0C2.S4-MMAF**					
	0.031 ± 0.005	0.0001	9.7780	10	0.024	Significant
	**H0C2.S5-MMAF**					
	0.775 ± 0.264	0.0009	4.6619	10	0.110	Significant
	**H0C2.S6-MMAF**					
	0.070 ± 0.011	0.0001	8.0127	10	0.024	Significant
**P:** the probability of success, **t:** the ratio of the departure of the estimated value of a parameter from its hypothesized value to its standard error, **df:** degree of freedom,						

Figure S5. Statistical significance for **c**ytotoxicity data (on the HER2 overexpressing cell line SK-BR-3) of conjugates H0C2.Sx-MMAF (x = 3 to 6) in comparison to reference 4D5.2-MMAF.
